# Endorsement and Constructive Criticism of an Innovative Online Reflexive Self-Talk Intervention

**DOI:** 10.3389/fpsyg.2019.01819

**Published:** 2019-08-06

**Authors:** Alexander T. Latinjak, Cristina Hernando-Gimeno, Luz Lorido-Méndez, James Hardy

**Affiliations:** ^1^School of Psychology and Education, University of Suffolk, Ipswich, United Kingdom; ^2^School of Health and Sport Sciences (EUSES), Universitat de Girona, Catalonia, Spain; ^3^Universitat Autònoma de Barcelona, Bellaterra, Spain; ^4^Institute for Psychology of Elite Performance, Bangor University, Bangor, United Kingdom

**Keywords:** self-esteem, anxiety, thoughts, self-regulation, inner speech, sports

## Abstract

This study prospectively followed the experiences of skilled athletes who were involved in an innovative reflexive self-talk online intervention targeting goal-directed self-talk. Four experienced female athletes between the ages of 20 and 40 years were invited to an initial interview, a 4-week intervention, and two post-intervention interviews. Two applied sport psychologists used an online Socratic questioning approach to encourage their athletes to describe challenging scenarios, think about their use of self-talk and its effectiveness, and explore alternative self-statements that could be used in future situations. Data were multi-sourced stemming from the psychologists, athletes, and third parties (e.g., coach). Three athletes completed the intervention, whereas one athlete withdrew prematurely, mainly because the Socratic questioning approach and the online mode of delivery did not meet her preferences. From the three athlete who had completed the intervention, there was endorsement and constructive criticism of the intervention and its online delivery mode. The intervention, largely due to the accompanying raised awareness of self-talk use and refined content, seemingly benefited a range of variables including emotions, motivation, and confidence, both inside and outside of the athletes’ sports life domain. Accordingly, this new type of online intervention warrants further consideration in the literature.

## Introduction

This study reports on a cognitive intervention that aims to change and strengthen athletes’ goal-directed self-talk in sports. This approach is aligned with interventions framed within cognitive therapy (Beck, 1976). Cognitive therapy emphasizes the role of internal dialog in influencing an individual’s subsequent feelings and behavior. According to Beck (1976), individuals are not always aware of their internal dialog, but they can learn to identify it, and, therefore, become able to monitor and, if necessary, replace automatic, emotion-filled thoughts. Cognitive-behavioral therapy (Meichenbaum, 1977) and rational emotive behavior therapy (Ellis, 1976) are two classical examples of cognitive therapy, which have successfully been applied to sport contexts (e.g., Neil et al., 2013; Turner and Barker, 2014) and in which self-talk plays a key role to cognitive change (Michie et al., 2013).

The terms *inner dialog* and *self-talk* were used by Beck (1976) and Meichenbaum (1977) mainly to refer to the critical inner voice that tends to encourage caution and self-doubt and can over time negatively impact upon self-esteem and self-worth (Palmer and Williams, 2013). In sport, the term *self-talk* is applied to a variety of processes that can occur simultaneously (Boudreault et al., 2018). To provide a conceptualization of self-talk that summarizes these processes, we describe it as follows: Self-talk takes form in verbalizations addressed to the self, overtly or covertly, characterized by interpretative elements associated to their content; and it either (a) reflects dynamic interplays between organic, spontaneous, and goal-directed, cognitive processes or (b) conveys messages to activate responses through the use of predetermined cues developed strategically, to achieve performance-related outcomes (Latinjak et al., 2019a).

In sport, self-talk interventions are usually beneficial for learning and performance and performance-related variables such as confidence or anxiety (Tod et al., 2011; Hatzigeorgiadis et al., 2014). However, in studies on the effects of self-talk, intervention protocols may be remarkably different (Latinjak et al., 2019a). Whereas traditional interventions focus on the effects of repetition of predetermined cue words (e.g., Hardy et al., 2015), some recent interventions aim to improve athletes’ rational self-regulatory skills by creating metacognitive knowledge (Brick et al., 2016). Changes in metacognition in these recent interventions result from repeated reflections on past organic self-talk (both spontaneous and goal-directed) and future use of self-instructions (Latinjak et al., 2016). This reflexive self-talk intervention aims to enhance the use of goal-directed self-talk, which is a controlled mental process deliberately employed toward solving a problem or making progress on a task (Latinjak et al., 2014).

According to a recent review on self-talk interventions (Latinjak et al., 2019a), there are three main differences between the traditional, strategic self-talk interventions, and the newly proposed reflexive intervention. First, the content of strategic self-talk interventions is typically pre-determined (Hardy, 2006), while the self-talk discussed in the reflexive interventions emerges from sport situations and is thus always self-determined. Second, the moment when the self-instructions are verbalized in strategic self-talk interventions is usually fixed to before or during the execution of the task. In reflexive self-talk interventions, participants must decide *in situ* when they want to use self-instructions. Third, while verbalizing self-instruction is essential in strategic self-talk interventions, the actual use of goal-directed self-talk is optional in the context of reflexive self-talk interventions. The result of a reflexive self-talk intervention could therefore even be to use less goal-directed self-talk, for example, to prevent ironic processes of mental control (Wegner, 1994).

Compared with the existing self-talk literature that deals intensively with research on interventions using predetermined cue words (Tod et al., 2011), the research with reflexive self-talk interventions (aka., goal-directed self-talk interventions) is still in its infancy (Latinjak et al., 2016, 2018). Nonetheless, diverse psychotherapeutic approaches [e.g., Rational Emotive Therapy (Ellis, 1976) and Cognitive-Behavior Modification (Meichenbaum, 1977)] previously applied effectively to the sports setting (Neil et al., 2013; Turner and Barker, 2014) serve as indirect support for the efficacy of reflexive self-talk interventions in sport. This is because, our reflexive self-talk intervention is similar to these psychotherapeutic approaches because it shares several core features. For instance, both cognitive-behavior approaches and reflexive self-talk interventions aim at making athletes conscious about their internal dialog, identifying automatic, emotion-filled thoughts, and when dysfunctional, replacing them with functional self-instructions (Beck, 1976; Latinjak et al., 2016). To this end, Socratic questioning (McArdle and Moore, 2012) is used to develop metacognitive skills that enable athletes to non-judgmentally observe their own thoughts, and subsequently think logically and empirically in order to challenge, correct, and replace them. In cognitive-behavioral therapy, Socratic questioning, which consists of asking a person a series of open-ended questions to help promote reflection, is considered useful for raising awareness and improving problem-solving thinking (Neenan, 2009).

A unique and contemporary aspect of the reflexive self-talk intervention presented in this study was the use of an online text-messenger service for the intervention. With an estimated 3 billion Internet users worldwide, the development of online interventions could be of considerable utility (Lane et al., 2016). To the best of the authors’ knowledge, only a single experiment has examined the effects of an online self-talk intervention in the performance context. Lane et al. (2016) examined the effects of strategic self-talk directed to outcome goals, process goals, instruction, and arousal-control, in a brief online intervention, on a competitive (non-sport) computer task. In their study, only self-talk directed to outcome and process goals helped participants’ performance. That said, at a more general level, their findings support the utility of the online modality to teach psychological skills.

Despite the lack of online interventions within the sport psychology literature, meta-data from other disciplines provide useful guidance. Specifically, research has emphasized the potential of online interventions in different areas of application including behavioral change, health, and clinical practice (e.g., Webb et al., 2010). An important matter in online interventions is related to the mode of delivery. Webb et al. (2010) performed a meta-analysis of online interventions, indicating that interventions that allowed for scheduled contact with an advisor showed significant effects, whereas interventions that provided automatic follow-up messages tended not to show significant effects. In addition, interventions using smart phones showed the biggest size effects among online interventions. Therefore, in our study, the use of automatized feedback was discarded and scheduled contact with an advisor via an online text-messenger service was preferred.

The present study included an innovative and longitudinal (4 weeks) self-talk intervention aimed at improving goal-directed self-talk using an online delivery format. The aim was to give a clear idea of what a successful reflexive self-talk intervention might look like and what variables should be considered to increase the likelihood of a satisfactory application. Aimed at applied practitioners, this study sought to provide the most detailed presentation of reflexive self-talk intervention procedures to date, as well as offer relevant and innovative guidance on adapting the standard procedures to the needs and preferences of individual athletes. In addition to investigating a novel form of self-talk intervention, the highly unusual but contemporary online format of the intervention is noteworthy. Our online delivery format has obvious scope and potential beyond just sport related self-talk; yet we are aware of very few published examples of online interventions in the sports psychology literature.

Overall, a 4-week reflexive self-talk online intervention was delivered and qualitative reports on implementation and perceived effects were collected. In order to gain a comprehensive understanding of the intervention, data from different sources (Tracy, 2010) were collected to compare different experiences of athletes, applied practitioners, and researchers. The experiences of athletes and psychologists were expected to reveal meaningful information for refining the intervention and highlight moderating factors that practitioners should consider when adjusting the intervention to their client’s specific needs.

## Materials and Methods

### Participants

#### Philosophical Orientation

Due to the investigation’s subjective focus, emphasizing the experiences of the participants, we adopted a constructivist epistemology enabling us to develop an appreciation of the lived experience and the identification of themes across our stakeholders. We assumed that there is no one knowledgeable truth and that knowledge involves a process of interpretation and the construction of individual knowledge representations (Jonassen, 1991). To this end, we collected data from a variety of sources – athletes, practitioners, and coaches – to provide a multifaceted understanding of a 4-week reflexive self-talk online intervention. Since our intervention was tailored to the individual circumstances of each athlete, it was expected that the experiences of our participants would be complex and dynamic. Therefore, a multiple single-case study approach was chosen as the most appropriate method. This approach is particularly useful for allowing analysis within and across individual cases that allow us to examine in detail the subjective experiences of individuals who are part of the intervention and to highlight similarities and differences between them. Accordingly, an interpretative phenomenological analysis was chosen to analyze the data, since it is relatively sensitive to exploring differences in experiences between participants (Sparkes and Smith, 2014).

#### The Athletes

To enhance the scope of our case study approach, four athletes were purposefully recruited for the study. We looked for athletes from different sports with different ages, different performance levels, but relatively large experience in their sports. All athletes participated in official competitions while the intervention took place. The four athletes between the ages of 20 and 40 years were involved in contact, choreographic and team sports, and participated in recreational and professional competitions. They all had over 7 years of experience and practiced over 10 h a week at the time of the intervention. Please note that for ethical reasons, we have changed the names of the participants and did not specify their exact sport and age.

#### The Psychologists

For this study, two novice sport psychologists with different of different ages (early 20s and early 40s) were selected. Both had <1-year experience in working as sport psychologists. The Psychologist 1 and the younger Psychologist 2 were graduated psychologists and specialists in sport and exercise psychology. In addition, Psychologist 1 had special training in Rational Emotive Behavior Therapy. Both participated in the design of the intervention and only after completion of the data collection, in the discussion of the results. Both worked at an elite sports academy with talented junior basketball players. They were selected for their interest in researching the use of online interventions and in providing self-talk interventions for athletes. Two athletes were randomly assigned to each psychologist. Psychologist 1 worked with Maria and Julia, while Psychologist 2 worked with Anna and Sandra.

### Procedures

#### Intervention Design

The main thrust of our intervention was based on Latinjak et al. (2016) reflexive self-talk intervention. Nonetheless, some experiences collected in that study and the decision to deliver the intervention via online text-messenger required further deliberation. To create the intervention protocol, the first author prepared a script that was discussed with the practitioners performing the intervention. After adapting and modifying the script, the intervention design was sent to the fourth author, who acted as critical friend in this study. Taking into account his comments, the first author elaborated the final protocol of the intervention.

#### Ethics and Athlete Sampling

After obtaining all necessary institutional permissions, athletes were selected, following recommendations about purposeful sampling in qualitative studies (Robinson, 2014). Accordingly, we defined a sample universe, we decided upon a sample size, through the conjoint consideration of epistemological and practical concerns, we selected convenience sampling as our sampling strategy, and we decided on contacting partner clubs and high-performance centers for sample sourcing. Suitable candidates were identified and contacted for an initial evaluation, via Skype, 1 week prior to the intervention. At the beginning of this interview, the athletes were informed about the procedures of the study and signed an informed consent form. Regarding confidentiality, athletes were informed that their names would be changed in the final report and none of their actual intervention discussions (i.e., text messages) would be published. In addition, the athletes were told that they would receive a copy of the summary of each interview to highlight sections that we should not quote in the article.

#### Initial Interview

One week before the intervention, the athletes participated in a brief interview conducted by a researcher independent of the intervention. The interview consisted of three parts. In Part 1, the athletes were asked personal descriptive questions (e.g., age, hours of practice, and best results in competitions). In Part 2, the athletes commented on their emotions, confidence, motivation, and thoughts in sport, and their corresponding self-regulation skills. In Part 3, the athletes were asked about their self-talk, in terms of frequency, typical things they say to themselves, and the effects of their self-talk on their sport participation.

Less than 48 h after the completion of the interview, each athlete was sent a transcript of her interview and a short summary, so that she could undertake modifications by rephrasing, eliminating, or adding ideas (if necessary). Once each athlete reflected on the interview transcriptions and the summary, the latter was sent to the psychologist who conducted the intervention.

#### Introductory Video

On day 1 of the intervention, the psychologists contacted each of the athletes sending them an introductory video via WhatsApp messenger. In this video, the leading researchers were introduced, and the general goals of the study were described. Specifically, the athletes were informed that this study aimed to test the effects of an online intervention on goal-directed thoughts in sport. Furthermore, the athletes were introduced to the idea of goal-directed self-talk, described as self-talk used intentionally to solve a problem or make progress on a task (Latinjak et al., 2019a). Several non-sport-related examples were offered in the video so as to inform but not bias participants.

After defining goal-directed self-talk, the general procedures of the intervention were outlined. That is, (a) all communications between you and the psychologist will take place in WhatsApp; (b) a typical session consists of you describing a problematic situation in your sport, reflect on your goal-directed self-talk in that situation, and evaluating potential alternative self-statements; (c) the aim of the intervention is to encourage you to reflect on your goal-directed self-talk, and so, the psychologist solely formulates questions and hardly ever provides answers; and (d) because research protocols have to be followed, other issues besides goal-directed self-talk cannot be discussed over the course of this intervention. Each of these points was accompanied by non-sport-related examples. After seeing the introductory video, the athletes were invited to formulate questions and they were informed that a psychologist would contact them within the next 3 days to start the intervention.

#### Intervention Sessions

**FIGURE 1:**
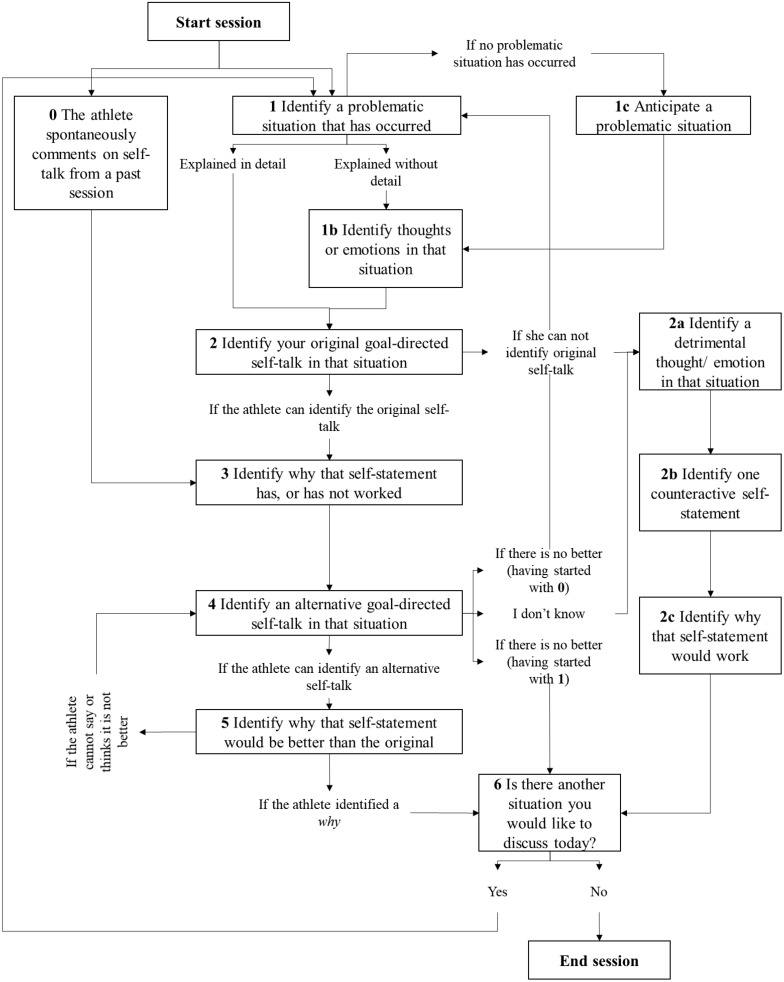
Flow chart representing the protocol in the reflexive self-talk online intervention for a single session.

During the intervention period, athletes were contacted every 3–4 days by their psychologist via WhatsApp, as scheduled by the athlete at the end of the previous session. Two days after the introductory video, the athletes were contacted for the first scheduled session. The psychologist opened the conversation asking the athlete “is it a good time to talk?” A typical session consisted of five consecutive main questions: (a) report a problematic situation that has occurred to you recently during training or competition; (b) what did you say to yourself in that situation to cope with your problems; (c) why did this statement help you to cope with the problems in that situation, or why did it not; (d) think of any alternative self-statement you could have used instead to self-regulate more effectively; and (e) why would this alternative statement be better compared with the original one to cope with the problems in the situation. Nonetheless, variations to this typical flow of the sessions were also foreseen (Figure 1).

#### Post-intervention Interview

In the week following the intervention, the athletes were contacted again via Skype by the same researcher who conducted the initial evaluation for a second interview. The post-intervention interview consisted of three parts. In Part 1, the athlete was asked to evaluate the general procedures of the intervention. Specific attention was paid to (a) the WhatsApp conversations and (b) the Socratic questioning approach. Both endorsement and constructive criticism were encouraged. In Part 2, the athletes were asked to reflect on changes they noted, or failed to notice (“have you hoped for some changes to take place, that haven’t taken place?”), with regard to the experience of and coping with emotions, confidence, motivation, and thoughts and attention. Finally, in Part 3, the athletes were asked to reflect on changes they noted, or failed to notice, regarding their use of self-talk as a self-regulation strategy. Again, less than 48 h after the interview, each athlete received a transcript of her interview and a short summary, so that modifications could be made, if necessary.

#### Third-Party Interviews

During the post-intervention interview, permission was requested to contact a significant person related to their sport (e.g., coach). The choice of that person was left to the athlete. Interviews with the third persons were conducted, within 2 weeks post-intervention, via Skype, by the same researcher who conducted the previous interviews with that athlete. During this interview, generic open-ended questions inquired into any changes in the athlete the coaches had observed during the past month.

#### Follow-Up Interview

Three months post-intervention, the athletes were contacted via Skype by the same researcher who conducted the previous evaluations, for a third interview. During the follow-up interview, the athletes were asked to reflect on changes in their sport, or even outside sport, that might (partly) be explained by the intervention conducted 3 months earlier. Some questions were also directed at exploring habits of self-reflection about self-talk participants might have acquired. Identical member checking procedures to those used previously were employed.

#### Psychologist’s Reflections

During the intervention, the psychologists followed a structured diary, enabling several intervention-control variables to be assessed: number of sessions per athlete (excluding the initial video), number of athlete messages, and a word count of athlete messages. Additionally, after the intervention had terminated, they were asked several questions regarding each athlete. In particular, they reflected on (a) the general functioning of the sessions, (b) any progresses they have noted, and (c) shortcomings or limitations of the interventions. Once this information was compiled and structured, the psychologists were given a copy and asked to reflect on the information correcting any mistakes, reformulating ideas, and adding missing information.

### Data Analysis

An interpretative phenomenological analysis was chosen to evaluate the data in this study. This approach enabled us to focus in depth on the interpretations and experiences of the athletes and psychologists. Furthermore, interpretative phenomenological analysis has recurrently been used in previous studies with small numbers of participants (Robinson, 2014).

In this study, the interpretative phenomenological analysis consisted of four steps that were consecutively performed on the transcripts of each athlete and psychologist. On an individual level, the analysis included (a) searching for themes by reading and re-reading all interviews and text-messages of the intervention and (b) identifying and labeling themes that characterize the experience and perceived effects of the intervention. On a group level, the two remaining steps consisted of (c) connecting the themes to make global sense of the athletes’ and psychologists’ reports and (d) producing a table for each participant (Tables 1–3) and two tables to summarize the reports of the athletes (Table 4) and psychologists (Table 5). For Sandra, no individual table was prepared, as she withdrew prematurely after 2 weeks of intervention. She just completed the initial interview and agreed after quitting to answer only a few questions regarding her withdrawal instead of the post-intervention interview.

**TABLE 1:** A summary of Maria’s initial, post-intervention, and follow-up interview.

	**Initial interview**	**Post-intervention interview**	**Follow-up interview**
Regarding emotions …	Maria **experienced frustration** (if you don’t progress and things go wrong, you get very frustrated) and **nervousness** (when I’m nervous my mind can’t calm down; I keep thinking while I perform the choreography). Generally, she feels a bit **excessively emotional** (I might also get overly emotional, I can start crying or suffer so much that it affects my performance).	Maria **gained consciousness** over her self-talk (… at some moments I was more conscious, and I tried to talk to myself, use alternative self-talk) and **improved her emotional coping** (I was able to cope with situations, especially when the situations were similar to those we had worked on). However, see **needed more time** (maybe I hadn’t enough time to assimilate it all; I think that in time I will cope better, but I hadn’t enough time yet and I still don’t know how to use it) because there still remains a **lack of awareness of some negative situations** (sometimes you are not conscious of the problematic situation and that you have to cope with it).	**The current use of self-talk:** I am talking to myself in several occasions, but much more positive (…); I have to say that I am much more conscient about my self-talk while [practicing my sport], but it is much more positive (.); no more “I can’t,” “you are doing it wrong” or “people are watching you”; instead, much more “come on, go!.”
Regarding motivation …	She reported about problems when **training technique** (if I go to class […] to improve technique, then it is true, that I might have more difficulties to motivate myself).	She detected a **better motivational self-regulation** (sometimes I am tired or nervous, and when I am tired my motivation lessens, and it [the intervention] helped me to motivate myself better in all these short moments when I decay). However, again, there remains a **lack of awareness of negative situations** (sometimes I am not motivated, I am tired, and I complain a lot and then I become aware; […] but not before I start complaining; I react a bit late).	**Thoughts about the intervention:** As I said, it was a very innovative experience, mainly because that is something no one is conscious about and within 1 month I became aware that I have an inner psychologist, an “inner I”, that I believe a lot in this inner I; she can talk to me, help me, but also haunt me (…); I think that it is great to discover the connection between your inner and outer I.
Regarding confidence …	She lacked confidence during **social comparisons** (it’s difficult because you constantly compare yourself to others or get turned down based on your CV or an audition) and after being **rejected** (it’s a no, no, no… all these rejections affect your confidence and constantly you ask yourself “am I good enough to do this).”	She might **care just a bit less** about the opinion of others (I think yes [there were changes], but we could not really test them yet… it helped me to care a bit less on what others might think, I have to be confident with my work) what helps her to **cope with sensations of being unprepared** (with regard to the auditions, when I thought I was not prepared I told myself ‘trust yourself’ […], in a different moment I would have thought no, you can’t, don’t go).	**Why did the intervention work:** I think it worked for me because I became more aware and able to redirect the inner conversation (…) to be more positive so that I can benefit, and not suffer, it.
Regarding cognitions …	**She had problems while being nervous** (when I am on the stage and I am nervous; my mind does not stop, and that goes against me).	She had perceived **great improvement** (I think here I have improved very much) partly because she managed to **reduce intrusive thoughts** (I had these intrusive thoughts on stage […] it didn’t happen to me again, and I am enjoying myself a lot because I can let go of myself and give 100% and if any thought appears I say “Maria, 100%” and it’s a good thought and I am very happy).	**The intervention had an effect outside sports:** I think in any aspect of life, work, love, life, family, sport … it can be applied everywhere.
Self-talk	She remembered **using self-talk** “during the warm-up, when you do routines and you have not to think (…); when I do push-ups or sit-ups.” However, she had experienced **a lack of control** over self-talk (I can’t [stop self-talk], it’s very hard for me). She remembered **positive effects** of self-talk when “you psych-up or you give strength to yourself; first I think ‘I am tired’ and then I think ‘come on, you can do it, you have to finish’.”	She **gained awareness** (I thought self-talk was something very conscious […], and then I realized that I might have thought things more unconsciously) and changed self-talk to be **more positive** (I tried to be more positive than before, when I had more negative self-talk; to psych up and not to drag me down). However, she knows it **isn’t perfect, yet** (I think I still can’t cope [with self-talk] 100%, to think always positive things), partly because **not all negative thoughts are conscious** (sometimes self-talk is unconscious, and I don’t realize I can cope with things using self-talk).	

**TABLE 2:** A summary of Anna’s initial, post-intervention, and follow-up interview.

	**Initial evaluation**	**Final evaluation**	**Follow-up interview**
Emotions	Anna mainly complained about **stress** (during some periods with more stress, sometimes I don’t eat correctly or when I am without a job … it’s harder to go to train; I have to make an effort, it’s easy to get distracted).	Anna feels that she can **cope better with fear** and, hence, she **stopped avoiding competitions** (before fear stopped me … before, competition? – no way; I wouldn’t even try to compete in my gym; when I got invited, I would say “how could I go to compete?” that decision was based on fear, thinking you are not worth anything … Now I go!). However, she knows that **there is work to do, still** (the intervention showed me the basics, I gained consciousness, but there still is a long way to go).	**Changes in awareness:** I am much more conscious, (…) because I also use it in other areas of my life, such as in academics or now that I am looking for a new job; Whenever I am more pessimistic I tell myself “it’s just something I am telling myself, and it does not help me at all.”
Motivation	She had problems with motivation **when she didn’t see progress** (I have been practicing [one sport] for 7 years, and still sometimes I ask myself how things can be that hard for me … techniques that do not work; so, where do I get my motivation from? From my colleagues?).	She noticed changes in motivation as **a correlate of competing** (my focus has changed; now it is no longer only [combat] practice, starting to compete was a change; it’s not about the trophies, it’s about the feeling I have after the competition, … despite the fear, I did it!).	**A remaining lack of belief in self-talk:** Sometimes I still need some confirmation (…) I tell myself again and again, but I need to see it become reality.
Confidence	For her, confidence was related to **visual aspects** of the tasks (confidence depends a lot on visual components in both [my sports]… to think if I can or I cannot, depends on the size of the weights, or the size of the opponent, or her facial traits).	With the intervention, **she got aware of her lack of confidence** (it’s like approaching the abyss, like a bird. It’s time to fly (…) you can’t stay in your comfort zone) and used self-talk to start **dealing with her lack of confidence** (I opened timidly my wings, the sensation is that I have hardly opened them but, I jumped; and I still am dwelling in satisfaction because I dared).	**Self-talk affects concentration:** I still have a tendency for mind wandering, but now I tell myself “it’s time to focus on the now.” I wander off many times, but I also return many times. It’s not like before, when circumstances made me return, now I make myself return.
Cognition	She had difficulties paying attention to **technical instructions** (I have some difficulties to maintain attention when receiving technical instructions; when I fight I focus almost automatically).	She noticed that she **gained awareness of her self-talk** (it’s a path you have opened to me, I start now, and I still have to keep on working; and, well, now I am much more conscious).	***Interviewer’s comment****: Once I turned off the microphone, I found Ruth incredibly thankful to the psychologist and the research team. She was looking forward to contacting the psychologist again, once the research had terminated.*
Self-talk	She reported **using self-talk** “mainly in [combats] when I am in advantage during a delicate moment, or when I am in a difficult situation, like being strangled.” She acknowledged a complete **lack of control** over self-talk (no, I am not in control) and **despair effects** (sometimes it works, it helps me to build upon my achievements; other times I can’t believe in my positive self-talk, and I can’t change the situation).	She claims to understand better **the importance of self-talk** (I see its very important what I was telling myself before; if I tell myself “you are so bad,” then when I fight I have to cope with that) and **how self-talk had influenced her decisions** (it was fantastic getting aware of unconscious decisions I had taken based on my negative self-talk). Now, she **transfers positive experiences to future challenges** (in situation in which I got conscious that my self-talk was positive, I kept these statements, as a tool, for other moments).	

**TABLE 3:** A summary of Julia’s initial, post-intervention, and follow-up interview.

**TE**	**Initial evaluation**	**Final evaluation**	**Follow-up interview**
Emotions	Julia found it hard to control her emotions when she experienced **external problems** (if I have external problems, they distract me, and I worry too much, it’s hard). One specific emotion, that affects her performance, is **sadness** (when I am sad I have to concentrate).	Julia has managed to adopt a **more flexible point of view** (I can look at things differently … I think I should approach things differently, from another point of view).	**Changes in emotions:** “Before I was ‘very nervous, like so scared’ and now it’s like ‘no, we can win and calm down, and if we don’t, nothing happens; it’s the way I talk to myself that helps me a lot; when I play I’m more courageous, and if I miss, I just continue.”
Motivation	She recognized that her **motivation can lead to nervousness** (sometimes motivation lead to nervousness, you know … when you motivate yourself so much; when you enter the pitch it’s like “I’m nervous” and sometimes I need to calm down and relax).	She noticed improvements in her motivation as **a consequence of changes in confidence** (it’s easier to motivate myself, because I am more confident now … it’s like a loop).	**Thoughts about the intervention:** “It went really well, all the program; for example the WhatsApp, it was key because in person I sometimes just think ‘I don’t know’; but, because I could write I had a moment to think and answer about thinks I couldn’t imagine to be potentially interesting; you asked questions that helped me to see things from a different point of view, and I often thought ‘I hadn’t realize that.”’
Confidence	She noticed a **lack of confidence** (I lack a lot of confidence and that’s a problem), specially **in comparison with others** (it makes me be worse than other just because they have more confidence). Moreover, she perceived that her **coach lacks confidence in her** (if the coach does not believe in me, it’s even harder for me).	For her, confidence was **the most important change** (it’s where I’ve see most changes, for the best, of course; I approach things differently, and that was like a door that opened to me). However, she still has to **test her confidence in competition** (I still have to put these things into action, so we’ll see), but she already **feels encouraged** (it psych’s you up, you know, to think “why haven’t I thought of it differently before,” it encourages you).	**The intervention had an effect outside sports:** “The whole program, the ‘what do you say to yourself’ and ‘what could you have said differently to yourself,’ can help you in all your life; maybe right now not, because I am doing really fine; or yes, also now; it can always help you, it’s so fantastic!”
Cognition	She experienced difficulties concentrating with **nervousness and fatigue** (when you are nervous you get tired really quickly, and then I lose concentration).	She perceived that the intervention helped her **to stay focused** (I concentrate much better; my mind does not disconnect), beginning with the **warm up** (I already start to focus during warm up, that helps later on).	
Self-talk	She remembered **using self-talk** “before the game, to concentrate and avoid anxiety, and after the game to analyze what happened. Her **control over self-talk** “depends on my physical state, when I am tired I can’t think properly.”	She gained **awareness of her self-talk** (now I have seen that all I say to myself are many things; I see how important these things are – what you say to yourself – to act one way or another), and her self-talk has helped her **to cope with her lack of confidence** (the confidence self-talk hadn’t work well before; now I know I need to take a different approach; I have seen that I shouldn’t tell myself “you can” and eliminate all the negative thoughts).	

**TABLE 4:** Athletes’ reflections about the online goal-directed self-talk intervention during the final interview.

**Evaluate**	**Maria**	**Anna**	**Julia**	**Sandra**
… the use of WhatsApp messenger:	**It fits into my daily routines:** …its good because, you can go on with your routine, you don’t have to go somewhere … its very contemporary … **It was a little impersonal:** … it is very important how you say things, and only writing is a bit cold … when talking via Skype, it is easier for me to express myself …	**It fits into my daily routines:** … It’s comfortable, you can answer from the train … **I thought on my own:** To understand myself (…) it’s easier when I am alone … **It was easier to write:** writing does not restrict me like being pressured by someone else … I read, then I think, it’s easier. **I missed gestures:** However, you need to find words, you can’t use gestures …	**It is easier to write:** … good, it’s always hard for me to explain things … it’s better to write … **I had more time:** it was great because when you made a question I had time to think about it … I could add things to my answer, and delete others … **Better than talking directly:** if we had talked, you know, I had just said “ok, yes …”	**I am unreliable when it gets to mobile phones:** … some days I forgot my phone at home and I didn’t get back until night … it happened 2 days we had to meet … **Skype would have been better:** … you should do it on skype … at least you see the others face, you see if it goes well or if she is lying, or if she had a terrible training …
… the use of questions instead of instructions:	**I depended on my own solutions and criteria:** … we are used, when being asked, to get feedback on your response … but then I thought maybe that is not necessary … things are neither good nor bad … it was really good because when asked (…) you come up with solutions you had previously not thought of … you see that you find solutions …	**I had to find my own solutions:** … since there were no answers I had to come up with them myself … answers can, unconsciously, bias me … **I didn’t feel evaluated**: I didn’t feel judged, I felt listened to, and, in my case, that worked very well … **I needed more feedback:** “I felt sometimes lost, in need for orientation or assessment …”	**Frustrating:** it’s a bit frustrating, … I would like to know what she thinks, from her point of view … but it’s like that, not too bad though.	**Repeated questions:** There was one day I told her (the psychologist) that I had trained very well, either way if I had told her that the training was terrible, she had asked me the exactly same question.
… the intervention generally:	**Interesting:** it’s a very interesting project … **Helps getting aware of self-talk**: … I gained much more consciousness than before … I was never aware of how some thoughts can affect you … they can change things in some situations.	**Positive experience:** Honestly, I had no idea how it would go, and still my expectancies were surpassed … **Noticeable changes:** I expected more questions and answers, and it was like that but the I noticed spectacular changes.	**Positive experience:** Good, very good. I didn’t expect anything … but it went very well. **Ran out of things to say:** I feel sorry that the last sessions I didn’t know any more what to talk about, but very good.	**It felt like talking to a Robot:** She (the psychologist) says something the first day, the second day the same, and the third, the fourth day I got tired … **I am just another message:** … you see that everyday it’s the same, I am just another WhatsApp…

**TABLE 5:** Overview of the length of the intervention, and basic reflections of the psychologists on their athletes’ intervention, progress, and limitations.

	**Athlete: Maria; Psychologist 1**	**Athlete: Anna; Psychologist 2**	**Athlete: Julia; Psychologist 1**	**Athlete: Sandra; Psychologist 2**
Number of sessions	Planned: 12; completed: 10; canceled: 2.	Planned: 12; completed: 12; canceled: 0.	Planned: 12; completed: 11; canceled: 1.	Planned: 13; completed: 6; canceled: 7.
Messages in completed sessions	115 sent to the participant (11.50 per session) and 110 received from the participant (11.00 per session).	169 sent to the participant (14.08 per session) and 156 received from the participant (13.00 per session).	155 sent to the participant (14.09 per session) and 155 received from the participant (14.09 per session).	83 sent to the participant (13.83 per session) and 78 received from the participant (13.00 per session)
General functioning of the interventions	Generally, the participant responded well to the established time tables and was actively involved in the intervention.	Some sessions were long, up to 90 min. It took us between 15 and 30 min just to find the first situation to work with.	The participant frequently changed the convened time schedule; yet, once the session had begun, she was answering without interruptions.	From the first session, the athlete did not meet at the convened hours. After 2 weeks, she stopped answering the messages I sent.
Progress made by the athlete as perceived by the psychologist	Initially, she gained awareness of her negative self-talk … later, she was able to reflect on it and turn it into positive thoughts … toward the end of the intervention, she was perfectly capable of identifying what she says and why it works or not … and she was able to look for alternatives in her self-talk …	We started talking about hypothetical situations in the beginning. After the sixth session, we discovered Pandora’s box, and we got to a more profound level after that. It took some time, but she got aware of her shame and fear while competing, and from there on she found ways to overcome them.	The participant had few problems identifying situations, emotions, thoughts, and self-talk and reflecting on the effects of the latter … I believe there was progress insofar as she still improved her ability to identify situations, the emotions and thoughts in that situation, and the effects of her self-talk.	There was no noticeable progress.
Shortcomings and limitations in the work with the athlete as perceived by the psychologist	… she recognized that sometimes it’s hard to believe in what she says, that the positive things she says are not always working, despite the search of alternative through thorough reflection …… specially when self-talk was used during strength and endurance tasks …	She had a hard time to identify what to talk about, and to connect with the feelings and thoughts in that situation. At the beginning, she had problems to reflect upon the situation, but after some session she was able to get to a conclusion faster.	… she has some difficulties believing in her self-talk, hence it often does not work … She often asked to talk about situations beyond the bounds of the intervention. The participant was not actively participating in the matches played during the intervention … hence, no situations around competitions emerged.	She never wanted to discuss any problematic situation that was really significant to her.

#### Establishing Confidence

Regarding the list of universal criteria for rigor in qualitative research (Tracy, 2010), in the present study a relativist approach was adopted (Sparkes and Smith, 2014). In the present study, the following criteria were included: the worthiness of the topic; the significant contribution of the work; rich rigor, that is, sampling diverse athletes, and psychologists to gather a variety of data from different sources that allow to understand a complex phenomenon; and the meaningful coherence of the research, indicating how well the study interlinks in terms of the aim, method, and results. Furthermore, the authors practiced self-reflexivity to consider how their perspectives influenced upon data collection and analysis. For example, having identified the first author’s potential bias in favor of the intervention’s effects, it was decided to have independent psychologists perform the intervention, to collect data from multiple sources, and to use multiple voices in the data analysis.

Regarding multiple sources of data and multiple voices in the analysis, these allow for different facets of problems to be explored to deepen our understanding. Besides the first author, the psychologists and the athletes, the fourth author of this study had served as a critical friend reviewing the intervention procedures and commenting critically on the final draft of the manuscript. In agreement with Cowan and Taylor (2016), the role of the critical friend was to encourage reflections upon, and exploration of, multiple and alternative explanations and interpretations of the data sampled in this study. For example, the critical friend was very important when we discussed the reasons why one of the athletes stopped the intervention prematurely. Based on his comments, we considered the relative lack of experience of the younger psychologist as a contributing factor. Furthermore, in order to facilitate a balanced perspective, efforts were made during in all interviews to capture and interpret both endorsements and constructive criticisms of the intervention.

## Results

In this section, the implementation and perceived effects of the intervention are described. First, we present the psychologists’ reports on the intervention and on the progress and limitations of their athletes. Subsequently, we summarize the evaluations athletes made during the post-intervention interviews. Furthermore, a third section outlines the specific outcomes of the intervention as interpreted by the researchers from the athletes’ interviews. Lastly, some testimony is offered, from third persons who were close to the participants during the intervention.

### The Psychologist’s Evaluation

#### Intervention Sessions

Two athletes, Maria and Anna, responded well to the established timetable (Table 5). Julia frequently changed the schedule and Sandra stopped the intervention after 2 weeks. Before canceling, Sandra had skipped several sessions and delayed others for several hours. Most sessions lasted between 20 and 45 min. With Anna, the sessions lasted much longer, up to 90 min. During the first sessions, she required up to 30 min to find a situation to discuss. After the third session, however, the sessions got noticeably shorter.

A total of 49 sessions were planned (12–13 per participant) and a total of 39 sessions were completed (6–12 per participant). All athletes but Sandra completed most of their scheduled sessions. Sandra only completed 6 out of 13 planned sessions. With regard to messages, 522 messages were sent from the psychologists to the athletes (83–169 per participant) and 499 messages were sent back from the athletes to the psychologists (78–156 per participant). See Table 5 for more detailed information concerning the sessions.

#### Content of Sessions

The athletes discussed a wide variety of idiosyncratic situations, including sport-specific situations, such as difficulties with a choreography (Maria), negative self-talk during competitions (Anna), problems concentrating (Julia), and situations in which things do not work out the way they were supposed to (Sandra). Furthermore, almost a third of the situations were not directly related to sport practice and performance. For instance, athletes talked about balancing free time and sports (Maria), and diet and injuries (Anna). Social conflicts, related to peers and coaches (Sandra), were also discussed. In all situations, athletes used self-talk to cope with, exclusively negative experiences such as anxiety, fear, stress, anger, shame, guilt, sadness, frustration, and pressure. The thoughts related to the situations were also negative; “I can’t stand the fatigue,” “I am not helping the team,” or, simply, “I can’t” are typical examples. Both negative experiences and thoughts occurred in competition, training, and outside of sport practice. The absence of positive emotions can be explained by the difficulties that athletes have in identifying positive experiences as detrimental for performance (Latinjak et al., 2016).

With a particular emphasis on athletes’ self-talk, two athletes, Maria and Julia, were able to discuss their self-talk in detail. Maria reported using instructions such as “Come on, concentrate, do it with energy” or “You can’t do everything 100%.” In her case, these instructions worked well, for example, when they helped her to accept the situation and not to see work as a loss of time. She also developed some new instructions during the intervention, such as, “Trust more in yourself and take your decisions” or “Think about the fun you will have tomorrow and that it was worth the effort.” With regard to Julia, she reported to have had used instructions such as “You can do better, proof it” or “Calm down.” These instructions helped if she managed to calm down. Nonetheless, often they ceased to work because she lost concentration or because some negative thoughts came back to debilitate her.

Skill-execution-related instructions, often studied in predetermined instructional self-talk interventions (Hardy et al., 2015), were not discussed by the athletes. Neither did the psychologists feel the need to direct the athletes through questions toward instructional statements. According to conscious processing hypothesis (Masters, 1992), modes of conscious control should mostly be used in early stages of learning, as they contrast with the typical automatic functioning of experts like the athletes in this study.

#### Evaluation of Athletes’ Progress

The psychologists noted a positive development in three of the athletes (Table 5). For example, according to the perceptions of Psychologist 1, Maria “gained awareness of her negative self-talk.” Once awareness was raised, “she was able to reflect on it and turn it into positive thoughts.” Finally, at the end of the intervention “she was able to look for alternatives in her self-talk.” The importance of awareness and motivation to change negative self-talk has received support in earlier studies in sports (Hardy et al., 2009b). In comparison, Julia even from the beginning, “had few problems identifying situations, emotions, thoughts, and self-talk, and reflecting on the effects of the latter.” For athletes with less awareness, it might be valuable to complete a self-talk diary ahead of their first session, to help them raise awareness and make the sessions run more efficiently. Another characteristic, related to awareness, is the athletes’ belief in their self-talk. While working with Maria, Psychologist 1 noticed that “she recognized that sometimes it’s hard for her to believe in what she says … .” Similarly, Julia “had some difficulties believing in her self-talk and, hence, it often does not work.” Previous studies have already focused on athletes’ belief in their self-talk (Hardy et al., 2009a). It therefore seems important to strengthen athletes’ beliefs in their inner voice, so that a change in self-talk content can be effective.

In the case of Sandra, who abandoned the intervention after only a few sessions, Psychologist 2 had not noted any progress. Sandra’s considerations indicate that it was the use of the online text-messenger service rather than the potential relative lack of experience of Psychologist 2, what may explain her withdrawal. Based on Sandra’s discontent with the intervention format, Psychologist 2 felt she “never wanted to discuss any problematic situation that was really significant to her.” Psychologist 2 also noted Sandra’s resistance to talk sincerely and to change her current self-regulation strategies (Zaltman and Duncan, 1977). Surely, Psychologist 2 lacked a bit of experience to better deal with resistance. However, it was mainly the intervention protocol that failed to include evidence-based techniques to deal with resistance (Hatcher, 2015). Because resistance is to be expected in cognitive-behavioral interventions, future studies on reflexive self-talk interventions should include strategic responses to optimize client experience and outcomes.

#### Advice for Practitioners

Based on their personal experiences, both psychologists formulated a series of proposals for applied practitioners. First, it is paramount to take your time to explore to some depth the situations that the athletes want to solve. It is those aspects they have not considered before that provide the best innovative solutions. Questions such as *why anxiety is making you perform worse* or *why others do not have the same problem* can help the athletes take an alternative perspective that leads to alternative goal-directed self-talk. Second, both psychologists agreed that a combination of text messages, voice recordings, and video-calls could be beneficial in applied practice.

### Athletes’ Reflections

#### Evaluation of the Intervention Format

The use of WhatsApp messenger received generally positive evaluations from Maria, Anna, and Julia, and negative evaluations from Sandra (Table 4). Generally, Maria and Anna acknowledged that the intervention fit very well into their daily routines. Nonetheless, this positive fit can be mediated by the tendency of athletes to use their mobile phones during the day. Sandra, on the contrary, frequently forgot her phone at home, where she did not return until very late every day.

The written messenger service format was rated positively because the athletes had time to think (Anna) and to write their answer, to change their answers, or to complete their answer before sending it (Julia). The disadvantages of the written messenger service were a lack of personal contact (Maria) and the absence of gestures (Anna). Although Maria and Sandra suggested that video chats might be an alternative to the written messenger service, for Julia it was the written format that had advantages over the video chat.

The Socratic questioning approach (McArdle and Moore, 2012) elicited disparate opinions among the athletes. Generally, Maria rated the questioning approach positively, Anna, both positive and negative, and Julia and Sandra rather negative. Both Maria and Anna acknowledged that the Socratic questioning approach required finding solutions on their own. For instance, Maria told us that “we are used to get feedback on our responses, but then I thought maybe that is not necessary; things are neither good nor bad ….” Additionally, Anna appreciated that she did not feel judged by the psychologist. Regarding the criticism of the Socratic questioning approach, both Anna and Julia found it frustrating not to receive any feedback from the psychologist. For instance, Anna explained that she “felt sometimes lost, in need for orientation or assessment.” For Sandra the problem was that the questions were repetitive. She reported that “one day I told her [Psychologist 2] that I had trained very well; either way if I had told her that the training was terrible, she would have asked me the exactly same question.” For context, please keep in mind the earlier argument on resistance in the relationship between Psychologist 2 and Sandra.

#### Overall Impression of the Intervention

When asked to critically evaluate the intervention, Maria, Anna, and Julia had a generally positive opinion (Table 4). For example, Julia told us that “I didn’t expect anything … but it went very well.” Anna specified that she “expected many questions and answers, and it was like that,” and then she “noticed spectacular changes.” Maria based her positive opinion on her increased awareness of self-talk. She reported that she “gained much more consciousness than before” when she “was never aware of how some thoughts can affect you … they can change things in some situations.” Sandra had a negative experience with the intervention. Specifically, the structured nature of the intervention did not meet her expectancies and preferences. She declared that “she [Psychologist 2] says something the first day, the same on the second and on the third day, and the fourth day I got tired.”

#### Follow-Up Interviews

In follow-up interviews, Maria, Anna, and Julia reported that some of the intervention effects on their self-talk were still noticeable (see Tables 1–3 for Maria, Anna, and Julia, respectively). Consistent with their post-intervention interviews, they kept noticing an enhanced awareness of self-talk. Maria told us that she “was much more aware of self-talk while [practicing my sport].” Moreover, she also detected that her self-talk was much more positive, insofar as “no more ‘I can’t’ or ‘you are doing it wrong’ or ‘people are watching you’.” Instead she used much more constructive statement, such as “come on, go!”

Furthermore, the three athletes acknowledged that for 3 months the changes in self-talk had a continuous impact on other performance-related variables. For example, Anna noted improvements in her concentration. She told us that she still had “a tendency for mind wandering” but now she told herself “it’s time to focus on the here and now.” Julia, in turn, noted improvements in her emotional control. She reported that before she was “very nervous, like so scared” and “now it’s like ‘no, we can win and calm down, and if we don’t, nothing happens’.” These comments were deemed positive, although it is unlikely that these changes can be attributed exclusively to the intervention. Be it as it may, the athletes’ comments provide support for the engagement with and acceptance of the athletes for the intervention, as all three see the intervention as the cause of positive changes in their sport.

According to Maria, Anna, and Julia, the intervention had positive long-term effects that were not restricted to sports because they identified changes in self-talk in other areas of life. Anna for example used self-talk consciously “in other areas of life, such as in academics or now when looking for a new job.” Julia believed that “the whole program, the ‘what do you say to yourself’ and ‘what could you have said differently to yourself’, can help you in all your life.”

It was found that even 3 months after the intervention, the athletes still evaluated the intervention as a positive experience. For Maria, it was important to discover “that I have a psychologist inside, an ‘inner I,’ that I believe a lot in this inner I, that she can talk to you, help you, or, on the contrary, haunt you.” More specifically, Julia remembered that “the WhatsApp (…) was a key point because in person I sometimes just think ‘I don’t know,’ but because I could write, you get your moment to think and answer … about things I couldn’t imagine to be potentially interesting.” On the basis of her experience with the intervention, Anna had even expressed her wish to continue working with her psychologist beyond the reflexive self-talk intervention. This suggests that online interventions for athletes can be a simple first step to commence working on psychological aspects, with positive experiences, leading to engagement in broader collaborations with sport psychologists.

### Interpreting Changes Across Athletes’ Pre- and Post-intervention Interviews

In this section, we present our interpretation of the pre- and post-intervention interviews (Table 4). This was possible only for Maria (Table 1), Julia (Table 2), and Anna (Table 3), as Sandra withdrew from the intervention. Sandra agreed to the final interview, but only to evaluate the intervention and briefly explain, from her point of view, what went wrong. Overall, our interpretation of the interviews suggests that the potential benefits of the intervention on performance is likely to result from the following sequence: the reflexive self-talk intervention (a) raises awareness of previous self-talk, (b) changes self-talk content, and (c) helps with performance-related variables like emotions, motivation, or confidence.

Generally, Maria, Julia, and Anna justified the positive effects of the intervention with an increase in metacognitive knowledge. Both Maria and Julia underlined that they gained awareness as they realized how they “might have thought things more unconsciously (Maria)” or that now they “have seen all that I say to myself, there are many things” and that they “see how important these things are (Julia).” Similarly, Anna reported that the intervention had helped her to understand the importance of self-talk (“I see how important the things I was telling myself before were”) and how self-talk had influenced her previous decisions (“It was fantastic, getting aware of unconscious decisions I had taken based on my negative self-talk”).

Alongside their increased awareness, all three athletes also noted positive experiences in refining their self-talk. For example, Maria changed her self-talk patterns as she “tried to be more positive than before, when I had more negative self-talk (…) to psych up and not to drag me down.” Anna even managed to transfer past successful self-talk experiences to future situations. She explained that “in situation in which I got conscious that my self-talk was positive, I kept these statements, as a tool, for other moments.” Julia managed to overcome a problem she had previously experienced when attempting to purposefully use self-talk: “The confidence self-talk hadn’t work well before. Now I know I need to take a different approach … I have seen that I shouldn’t tell myself ‘you can’ and eliminate all the negative thoughts.” Julia now focuses her self-talk on finding solutions for her problems instead of increasing confidence. She understood that confidence is a consequence of having found viable solutions. This last quote shows a connection between the reflexive self-talk intervention and the coping literature, where studies have found that female athletes use emotion-oriented rather than problem-oriented coping strategies (Crocker et al., 2015), although the latter generally lead to better outcomes (Nicholls and Polman, 2007).

The awareness and the changes of self-talk were associated to improvements in performance-related variables. Anna, for example, detected progress in her emotion-regulation. She reported that “before fear stopped me (…); I wouldn’t even try to compete in my gym (…); Now I go!” Maria also described positive changes in her motivation, as the intervention helped her “to motivate myself better in all these short moments when I decay.” For Julia, the most important change was related to her confidence. She told us that confidence is “where I’ve seen most changes, and for the good, of course; (…) I approached things differently, and that was like a door that opened.”

Notwithstanding, the athletes also recognized that further changes in the awareness and content of self-talk were required to better self-regulate. Maria, for example, admitted that “I think I still can’t cope [with self-talk] 100%, to think always positive things.” Specifically, she told us that “sometimes I am unaware of self-talk, and I don’t comprehend that I could cope with things using self-talk.” Maria and Anna argued that they had needed more time. For instance, Maria told us that she “had not enough time to assimilate it all,” and Anna recognized that “the intervention showed me the basics, I gained consciousness, but there still is a large way to go.” On the positive side, Maria and Anna were keen to continue the intervention even 3 months after it had ended.

### Third-Party Reflections on the Intervention

Two athletes, Maria and Julia, gave us permission to contact a significant person in their sport environment to corroborate the effects of the intervention. On the contrary, Anna did not allow us to contact anyone close to her. She preferred “those few people, who know me well enough to evaluate any changes, not to be involved with the intervention.” Marc, Maria’s training partner noted meaningful changes that confirm her reports on enhanced self-motivation. Before the intervention, “Maria tended to react negatively to challenges and mistakes,” Marc explained. She “was the first to say things like ‘I can’t do it’,” what “had effects on others, because if you have someone telling your constantly ‘I can’t, I can’t’, (…) well, we have to be positive.” After the intervention, Marc noticed that “she lets herself go more, she’s focused on enjoying herself.” In summary, Marc saw her “more motivated, more optimistic.” Julia’s coach also corroborated the positive changes his pupil had noticed in her confidence. Oriol explained that “she started to show a lot of confidence, she finished off plays, and she took responsibility in very important moments during the games, something anyone wouldn’t do without confidence.”

## Discussion

In this study, an online version of a novel reflexive self-talk intervention (Latinjak et al., 2016) was implemented, and experiences of its application and perceived effects were gathered over a prolonged period of time from multiple sources. The online text-messenger format received both approval and criticism. The potential beneficial effects of the intervention seem to be based on (a) raised awareness of previous self-talk, (b) refined self-talk content, and (c) effects on performance-related variables such as emotions, motivation, or confidence. The intervention was rated positively by three of the four participants, who noted positive effects both in sport and outside their sport.

Self-awareness has been identified as a fundamental psychological skill for athletes and one of four fundamental components of effective self-regulation (Vealey, 2007; Heatherton, 2011). Awareness is also connected to metacognition, insofar as Zimmerman (2000, p. 65) defined metacognition as “the awareness of and knowledge about one’s own thinking.” This is relevant as metacognition is an essential component of self-regulation and its primary functions are to monitor and control the thoughts and actions required for sport performance (Brick et al., 2016). As a result, it is thought that the effects of our online reflexive self-talk intervention are accompanied by an improvement in metacognition, which is caused by the reflection and planning of self-talk.

According to Zinsser et al. (2006) it is possible that this heightened awareness can cause athletes to change their self-talk patterns in order to improve sport performance. However, it is likely that self-talk does not affect performance *per se*, but through changes in performance-related mechanisms (Galanis et al., 2016). In the present study, the participants reported benefits in terms of concentration, confidence, motivation, and emotional control. This is in line with goal-directed self-talk categories that have been uncovered in previous studies. Boudreault et al. (2018) described, as an example, motivational and emotion control functions of goal-directed self-talk, which reflect many of the participants’ comments on the outcomes of the present intervention. Likewise, concentration and confidence-oriented statements are among the most replicated findings in the research on goal-directed self-talk (e.g., Latinjak et al., 2014, 2019b). However, all of these studies were descriptive and therefore cannot establish a causal link between goal-directed self-talk and performance-related variables. In order to find inferential evidence, one must refer to research with strategic self-talk interventions (e.g., Hardy et al., 2015), in which self-talk is however far less self-determined. These studies indirectly support the findings of this project as they demonstrate that self-talk can have a positive effect on concentration, confidence, motivation, and emotional control (e.g., Tod et al., 2011), and that changes in these factors may partly explain how self-talk improves sport performance (Hatzigeorgiadis et al., 2014).

### Issues Relevant to Applied Practice

Several considerations are important before utilizing the reflexive self-talk intervention. These relate to expectancies and/or preferences of athletes when working with sport psychologists. First, when opting for self-talk interventions, cognitive processing preference should be considered. For instance, it was apparent that Anna had a very little preference for self-talk, and this coincided with her being relatively unaware of her inner dialog and how her inner dialog affected her sport participation. Conversely, Julia showed a strong preference for using self-talk and was, thus, relatively conscious of her self-talk even prior to the intervention. Nevertheless, there remains little evidence about the impact of cognitive processing preference on the use of self-talk and its effects (for an exception see, Thomas and Fogarty, 1997). In the present case, cognitive processing preference might explain why the intervention was considered too short by Anna, and why the time gap between sessions was perceived too narrow by Julia, who eventually ran out of self-talk to discuss. With regards to further individual differences and their effect on self-talk use, it is noteworthy in views of the present study that previous studies found differences between males and females (Latinjak et al., 2017; Ada et al., 2019).

Second, applied practitioners need to decide whether to use the traditional strategic or the innovative reflexive self-talk intervention. Strategic self-talk interventions are simpler and lead to fixed self-talk plans to be used at particular instances to deal with fixed and specific performance issues (e.g., see also the IMPACT-ST model by Hatzigeorgiadis et al., 2014). Alternatively, more self-determined interventions, such as the reflexive self-talk intervention (Latinjak et al., 2016), are more malleable and less controlled, and aim to improve metacognitive skills. Within reflexive self-talk interventions, a Socratic questioning approach is indispensable. Maria and Anna evaluated the Socratic questioning approach positively, whereas Julia and Sandra would have preferred more guidance and assurance. Both psychologists advocated the use of scaffolding for applied practice. Scaffolding is when the psychologist provides temporary support to the athletes to gain a deeper understanding of their psychological challenges and the role of self-talk as a psychological skill (James et al., 2010). In this context, athletes may first become familiar with basic aspects of cognitive therapy. Such psychoeducation on the influence of thought on emotions and behavior has proven to be important for cognitive interventions (Kazantzis et al., 2018). Along these lines, guidelines on the use of feedback should also be included in the intervention protocol. In time, the scaffolds used at the beginning of the intervention would gradually be removed as athletes progressively gain an understanding of the reflective task, they are to perform. Overall, it will be important in future studies to add detailed guidelines for the provision of scaffolding to the intervention procedures, and thus successfully overcome challenges such as resistance. This information could be particularly useful for relatively inexperienced practitioners such as the psychologists who participated in this study.

Third, use of an online intervention delivery format or traditional face-to-face sessions (Latinjak et al., 2016) is worthy of further consideration. In the present study, the athletes communicated with their psychologist by mobile phone. This format was chosen because online interventions, administered via mobile phones, will become more and more accessible to different populations as the rate of ownership of smart phones rises [e.g., in the United Kingdom, from 60% ownership in 2013 to 80% by the end of 2017 (García et al., 2016)]. However, in practice, athletes should feel comfortable with mobile phones for this delivery option to be viable. Sandra, for instance, used her discomfort with mobile phones to partially explain her withdrawal from the intervention. To contextualize the experiences reported in this study, it should also be noted that demographic studies have shown that men and women use online messenger services differently (Rosenfeld et al., 2018).

Fourth, having chosen the online format, the applied practitioner is still left with the choice of written or verbal communication. Julia explicitly acknowledged the importance of the written response format as it allowed her to take her time and to write and rewrite her answers. However, Maria and Sandra would have preferred video chat in combination with the text-messenger application. Our decision to employ a text-based format was informed by Pennebaker’s (1997) work investigating expressive writing. Nonetheless, Pennebaker and Seagal (1999) reported that expressive writing and expressive talking should have comparable effects. Based on the available evidence, practitioners may consider taking the athletes’ preference of one or the other communication format into account.

### Methodological Considerations

In this investigation, member reflecting was used as a means to enhance rigor in the qualitative research design. Member reflections were considered to ensure the manuscript would reflect the subjective experience of both athletes and psychologists. Hence, in this study epistemological constructivism and ontological relativism were preferred over ontological realism (Smith and McGannon, 2017). This approach was aligned with the present goal to collect qualitative evidence about the delivery and perceived effects of our online reflexive self-talk intervention. Future studies, however, should also be grounded within an ontological realism framework; that is, gather objective evidence to confirm the effects of the reflexive self-talk intervention in sports, and its broader effects beyond the boundaries of sport. With regard to future research, we also recommend testing the application and effects of reflexive self-talk interventions in non-sport contexts. The use of goal-directed self-talk is common in a variety of contexts, including but not limited to physical activity and academic and professional activities.

## Conclusion

This innovative study provides a detailed insight into an online version of a reflexive self-talk intervention. The steps of the intervention protocol and involvement of the client is best summarized by: (a) a description of recurrent problematic situations in and around sports, (b) reflections on situation-specific goal-directed self-talk and its effectiveness, and (c) the development of alternative statements that can be used in future situations. The online text-messenger may be beneficial as it allows athletes (a) to engage with the intervention when it best suits them, at any location of their convenience, (b) to take as much time as they required to reflect on the intervention questions, and (c) give their concise responses in a written format. The potential beneficial effects of the intervention seem to be based on; (a) raised awareness of previous self-talk, (b) refined self-talk content, and (c) effects on performance-related variables like emotions, motivation, or confidence. The intervention protocol displayed in Figure 1 can be taken as a starting point for applied practice. Yet, some sections of the protocol would benefit from further development. To improve the protocol, applied practitioners should: (a) integrate guidelines for dealing with resistance, (b) consider using scaffolding during the initial sessions, and (c) combine text messenger and video chat options. The increasingly popular voice recording function in text-messenger applications is another suitable option.

The intervention described in this study is very different to the traditional strategic self-talk interventions investigated over the last three decades yielding generally positive effects for sport performance (Tod et al., 2011). Whereas strategic self-talk interventions targeted changes in psychological processes, such as confidence or emotions (Hatzigeorgiadis et al., 2009), this new reflexive self-talk intervention aims to enhance metacognitive knowledge. Athletes are encouraged to learn about themselves, and to use this knowledge to better self-regulate both in and outside of their sport. Hence, this is a self-talk intervention developed and applied in sport, with potential beneficial effects for the athlete in other areas of life. Our highly unusual online delivery format also serves as a reminder to both practitioners and researchers of the need to be responsive to changes in, and athletes’ use of, information technology. This intervention represents one of very few in the sports psychology literature that embraces an online methodology.

## Data Availability

The datasets generated for this study are available on request to the corresponding author.

## Ethics Statement

This study was carried out in accordance with the recommendations of the University of Suffolk Research Ethics Committee, with written informed consent from all subjects. All subjects gave written informed consent in accordance with the Declaration of Helsinki. The protocol was approved by the University of Suffolk Research Ethics Committee.

## Author Contributions

AL and JH designed the study. AL, CH-G, and LL-M collected the data and analyzed the data. CH-G and LL-M ran the intervention. AL wrote the manuscript. JH made extensive comments on the manuscript. CH-G and LL-M provided feedback on the manuscript.

## Conflict of Interest Statement

The authors declare that the research was conducted in the absence of any commercial or financial relationships that could be construed as a potential conflict of interest.

## References

[B1] Ada E. N. Comoutos N. Karamitrou A. Kazak Z. (2019). Relationships between dispositional flow, motivational climate, and self-talk in physical education classes. *Phys. Educ.* 76 357–384. 10.18666/TPE-2019-V76-I2-8419

[B2] Beck A. T. (1976). *Cognitive Therapy and the Emotional Disorders.* New York, NY: New American Library.

[B3] Boudreault V. Trottier C. Provencher M. D. (2018). Investigation of the self-talk of elite junior tennis players in a competitive setting. *Int. J. Sport Psychol.* 49 386–406.

[B4] Brick N. E. MacIntyre T. E. Campbell M. J. (2016). Thinking and action: a cognitive perspective on self-regulation during endurance performance. *Front. Physiol.* 7:159. 10.3389/fphys.2016.00159 27199774 PMC4847621

[B5] Cowan D. Taylor I. M. (2016). ‘I’m proud of what I achieved; I’m also ashamed of what I done’: a soccer coach’s tale of sport, status, and criminal behaviour. *Qual. Res. Sport Exerc. Health* 8 505–518. 10.1080/2159676X.2016.1206608

[B6] Crocker P. R. E. Tamminen K. A. Gaudreau P. (2015). “Coping in sport,” in *Contemporary Advances in Sport Psychology: A Review*, eds Hanton S. Mellalieuc S. (New York, NY: Routledge), 28–67.

[B7] Ellis A. (1976). *Reason and Emotion in Psychotherapy.* New York, NY: Lyle Stuart.

[B8] Galanis E. Hatzigeorgiadis A. Zourbanos N. Theodorakis Y. (2016). “Why self-talk is effective? Perspectives on self-talk mechanisms in sport,” in *Sport and Exercise Psychology Research: From Theory to Practice*, eds Raab M. Wylleman P. Seiler R. Elbe A.-M. Hatzigeorgiadis A. (London: Elsevier).

[B9] García B. Welford J. Smith B. (2016). Using a smartphone app in qualitative research: the good, the bad and the ugly. *Qual. Res.* 16 508–525. 10.1177/1468794115593335

[B10] Hardy J. (2006). Speaking clearly: a critical review of the self-talk literature. *Psychol. Sport Exerc.* 7 81–97. 10.1016/j.psychsport.2005.04.002

[B11] Hardy J. Begley K. Blanchfield A. W. (2015). It’s good but it’s not right: instructional self-talk and skilled performance. *J. Appl. Sport Psychol.* 27 132–139. 10.1080/10413200.2014.959624

[B12] Hardy J. Oliver E. Tod D. (2009a). “A framework for the study and application of self-talk in sport,” in *Advances in Applied Sport Psychology: A Review*, eds Mellalieu S. D. Hanton S. (London: Routledge), 37–74.

[B13] Hardy J. Roberts R. Hardy L. (2009b). Awareness and motivation to change negative self-talk. *Sport Psychol.* 23 435–450. 10.1123/tsp.23.4.435

[B14] Hatcher R. L. (2015). Interpersonal competencies: responsiveness, technique, and training in psychotherapy. *Am. Psychol.* 70 747–757. 10.1037/a0039803 26618963

[B15] Hatzigeorgiadis A. Zourbanos N. Latinjak A. T. Theodorakis Y. (2014). “Self-talk,” in *Routledge Companion to Sport and Exercise Psychology* eds Papaioannou A. Hackfort D., (New York, NY: Routledge), 372–386.

[B16] Hatzigeorgiadis A. Zourbanos N. Mpoumpaki S. Theodorakis Y. (2009). Mechanisms underlying the self-talk–performance relationship: the effects of motivational self-talk on self-confidence and anxiety. *Psychol. Sport Exerc.* 10 186–192. 10.1016/j.psychsport.2008.07.009

[B17] Heatherton T. F. (2011). Neuroscience of self and self-regulation. *Annu. Rev. Psychol.* 62 363–390. 10.1146/annurev.psych.121208.131616 21126181 PMC3056504

[B18] James I. A. Morse R. Howarth A. (2010). The science and art of asking questions in cognitive therapy. *Behav. Cogn. Psychother.* 38 83–93. 10.1017/S135246580999049X 19922709

[B19] Jonassen D. H. (1991). Objectivism versus constructivism: do we need a new philosophical paradigm? *Educ. Technol. Res. Dev.* 39 5–14. 10.1007/BF02296434

[B20] Kazantzis N. Luong H. K. Usatoff A. S. Impala T. Yew R. Y. Hofmann S. G. (2018). The processes of cognitive behavioral therapy: a review of meta-analyses. *Cogn. Ther. Res.* 42 349–357. 10.1007/s10608-018-9920-y

[B21] Lane A. M. Totterdell P. MacDonald I. Devonport T. J. Friesen A. P. Beedie C. J. (2016). Brief online training enhances competitive performance: findings of the BBC Lab UK psychological skills intervention study. *Front. Psychol.* 7:413. 10.3389/fpsyg.2016.00413 27065904 PMC4811866

[B22] Latinjak A. T. de Las Heras B. Sacot A. Fernandez D. Robinson D. Lane A. M. (2018). Effects of reflection to improve goal-directed self-talk on endurance performance. *Sports* 6:55. 10.3390/sports6020055 29910359 PMC6027548

[B23] Latinjak A. T. Font-Lladó R. Zourbanos N. Hatzigeorgiadis A. (2016). Goal-directed self-talk interventions: a single-case study with an elite athlete. *Sport Psychol.* 30 189–194. 10.1123/tsp.2015-0120

[B24] Latinjak A. T. Hatzigeorgiadis A. Comoutos N. Hardy J. (2019a). Speaking clearly …10 years on: the case for an integrative perspective of self-talk in sport. *Sport Exerc. and Perform. Psychol.* 10.1123/tsp.2015-0120

[B25] Latinjak A. T. Torregrossa M. Comoutos N. Hernando-Gimeno C. Ramis Y. (2019b). Goal-directed self-talk used to self-regulate in male basketball competitions. *J. Sport Sci.* 37 1429–1433. 10.1080/02640414.2018.1561967 30616448

[B26] Latinjak A. T. Hatzigeorgiadis A. Zourbanos N. (2017). Goal-directed and spontaneous self-talk in anger-and anxiety-eliciting sport-situations. *J. Appl. Sport Psychol.* 29 150–166. 10.1080/10413200.2016.1213330

[B27] Latinjak A. T. Zourbanos N. López-Ros V. Hatzigeorgiadis A. (2014). Goal-directed and undirected self-talk: exploring a new perspective for the study of athletes’ self-talk. *Psychol. Sport Exerc.* 15 548–558. 10.1016/j.psychsport.2014.05.007

[B28] Masters R. S. W. (1992). Knowledge, (k)nerves and know-how: the role of explicit versus implicit knowledge in the breakdown of a complex motor skill under pressure. *Br. J. Psychol.* 83 343–358. 10.1111/j.2044-8295.1992.tb02446.x 17470398

[B29] McArdle S. Moore P. (2012). Applying evidence-based principles from CBT to sport psychology. *Sport Psychol.* 26 299–310. 10.1123/tsp.26.2.299

[B30] Meichenbaum D. (1977). *Cognitive-Behaviour Modification: An Integrative Approach.* New York, NY: Plenum Press.

[B31] Michie S. Richardson M. Johnston M. Abraham C. Francis J. Hardeman W. (2013). The behavior change technique taxonomy (v1) of 93 hierarchically clustered techniques: building an international consensus for the reporting of behavior change interventions. *Ann. Behav. Med.* 46 81–95. 10.1007/s12160-013-9486-6 23512568

[B32] Neenan M. (2009). Using Socratic questioning in coaching. *J. Ration. Emot. Cogn. Behav. Ther.* 27 249–264. 10.1007/s10942-007-0076-z

[B33] Neil R. Hanton S. Mellalieu S. D. (2013). Seeing things in a different light: assessing the effects of a cognitive-behavioral intervention upon the further appraisals and performance of golfers. *J. Appl. Sport Psychol.* 25 106–130. 10.1080/10413200.2012.658901

[B34] Nicholls A. R. Polman R. C. (2007). Coping in sport: a systematic review. *J. Sports Sci.* 25 11–31. 10.1080/02640410600630654 17127578

[B35] Palmer S. Williams H. (2013). “Cognitive behavioral approaches,” in *The Wiley-Blackwell Handbook of the Psychology of Coaching and Mentoring*, eds Passmore J. Peterson D. B. Feire T. (Oxford: John Wiley & Sons), 319–338.

[B36] Pennebaker J. W. (1997). Writing about emotional experiences as a therapeutic process. *Psychol. Sci.* 8 162–166. 10.1111/j.1467-9280.1997.tb00403.x

[B37] Pennebaker J. W. Seagal J. D. (1999). Forming a story: the health benefits of narrative. *J. Clin. Psychol.* 55 1243–1254. 10.1002/(sici)1097-4679(199910)55:10<1243::aid-jclp6>3.0.co;2-n 11045774

[B38] Robinson O. C. (2014). Sampling in interview-based qualitative research: a theoretical and practical guide. *Qual. Res. Psychol.* 11 25–41. 10.1080/14780887.2013.801543

[B39] Rosenfeld A. Sina S. Sarne D. Avidov O. Kraus S. (2018). WhatsApp usage patterns and prediction of demographic characteristics without access to message content. *Demogr. Res.* 39 647–670. 10.4054/demres.2018.39.22

[B40] Smith B. McGannon K. R. (2017). Developing rigor in qualitative research: problems and opportunities within sport and exercise psychology. *Int. Rev. Sport Exerc. Psychol.* 11 101–121. 10.1080/1750984X.2017.1317357

[B41] Sparkes A. C. Smith B. (2014). *Qualitative Research Methods in Sport, Exercise and Health: From Process to Product.* London: Routledge.

[B42] Thomas P. R. Fogarty G. J. (1997). Psychological skills training in golf: the role of individual differences in cognitive preferences. *Sport Psychol.* 11 86–106. 10.1123/tsp.11.1.86

[B43] Tod D. Hardy J. Oliver E. J. (2011). Effects of self-talk: a systematic review. *J. Sport Exerc. Psychol.* 33 666–687. 10.1123/jsep.33.5.666 21984641

[B44] Tracy S. J. (2010). Qualitative quality: eight “big-tent” criteria for excellent qualitative research. *Qual. Inq.* 16 837–851. 10.1177/1077800410383121

[B45] Turner M. J. Barker J. B. (2014). Using rational emotive behavior therapy with athletes. *Sport Psychol.* 28 75–90. 10.1123/tsp.2013-0012

[B46] Vealey R. S. (2007). “Mental Skills Training in Sport,” in *Handbook of Sport Psychology*, 3a Edn, eds Tenenbaum G. Eklunt R. C. (New Jersey, NJ: John Wiley & Sons, Inc.), 287–309.

[B47] Webb T. L. Joseph J. Yardley L. Michie S. (2010). Using the internet to promote health behavior change: a systematic review and meta-analysis of the impact of theoretical basis, use of behavior change techniques, and mode of delivery on efficacy. *J. Med. Internet Res.* 12:e4. 10.2196/jmir.1376 20164043 PMC2836773

[B48] Wegner D. M. (1994). Ironic processes of mental control. *Psychol. Rev.* 101 34–52. 10.1037/0033-295X.101.1.34 8121959

[B49] Zaltman G. Duncan R. (1977). *Strategies for Planned Change.* New York, NY: John Wiley & Sons.

[B50] Zimmerman B. J. (2000). Self-efficacy: an essential motive to learn. *Contemp. Educ. Psychol.* 25 82–91. 10.1006/ceps.1999.1016 10620383

[B51] Zinsser N. Bunker L. Williams J. (2006). “Cognitive techniques for building confidence and enhancing performance,” in *Applied Sport Psychology: Personal Growth to Peak Performance*, 5th Edn, ed. Williams J. M. (New York, NY: McGraw Hill), 349–381.

